# Continuous Submicron Particle Separation Via Vortex-Enhanced Ionic Concentration Polarization: A Numerical Investigation

**DOI:** 10.3390/mi13122203

**Published:** 2022-12-12

**Authors:** Rasool Dezhkam, Hoseyn A. Amiri, David J. Collins, Morteza Miansari

**Affiliations:** 1 Micro+Nanosystems and Applied Biophysics Laboratory, Department of Mechanical Engineering, Babol Noshirvani University of Technology, Babol 4714873113, Iran; 2 Department of Cancer Medicine, Cell Science Research Center, Royan Institute for Stem Cell Biology and Technology, ACECR, Isar 11, Babol 4713818983, Iran; 3 Department of Mechanical Engineering, Sharif University of Technology, Tehran 113658639, Iran; 4 Department of Biomedical Engineering, University of Melbourne, Melbourne, VIC 3010, Australia; 5 The Graeme Clark Institute, The University of Melbourne, Melbourne, VIC 3010, Australia

**Keywords:** ion concentration polarization (ICP), DC dielectrophoresis (DEP), water purification, particle manipulation, virus detection, disease extraction

## Abstract

Separation and isolation of suspended submicron particles is fundamental to a wide range of applications, including desalination, chemical processing, and medical diagnostics. Ion concentration polarization (ICP), an electrokinetic phenomenon in micro-nano interfaces, has gained attention due to its unique ability to manipulate molecules or particles in suspension and solution. Less well understood, though, is the ability of this phenomenon to generate circulatory fluid flow, and how this enables and enhances continuous particle capture. Here, we perform a comprehensive study of a low-voltage ICP, demonstrating a new electrokinetic method for extracting submicron particles via flow-enhanced particle redirection. To do so, a 2D-FEM model solves the Poisson–Nernst–Planck equation coupled with the Navier–Stokes and continuity equations. Four distinct operational modes (Allowed, Blocked, Captured, and Dodged) were recognized as a function of the particle’s charges and sizes, resulting in the capture or release from ICP-induced vortices, with the critical particle dimensions determined by appropriately tuning inlet flow rates (200–800 [µm/s]) and applied voltages (0–2.5 [V]). It is found that vortices are generated above a non-dimensional ICP-induced velocity of $U^{*}=1$, which represents an equilibrium between ICP velocity and lateral flow velocity. It was also found that in the case of multi-target separation, the surface charge of the particle, rather than a particle’s size, is the primary determinant of particle trajectory. These findings contribute to a better understanding of ICP-based particle separation and isolation, as well as laying the foundations for the rational design and optimization of ICP-based sorting systems.

## 1. Introduction

The separation and isolation of micron and submicron scale particles are necessary for a broad range of applications, from desalination to environmental and biomedical research [1]. It can be used, for instance, to filter viruses/bacteria or hazardous materials from water [2,3]. Micromanipulation techniques can accomplish this via particle separation [4] and/or trapping [5] approaches. In the context of microfluidic devices, manipulation techniques can be in lab-on-chip platforms [6], with multiple processes occurring in a single device. Such microfluidic devices offer the ability to utilize physical effects and phenomena for micro-manipulation that would not be feasible at larger length scales [7,8]. Microfluidic devices have accordingly been investigated for a wide range of applications based on differential micromanipulation, including desalination [9], on-chip analysis [10], disease detection [11,12], and bio-particle separation [13,14]. Accordingly, several principles of separation have been explored using passive and active approaches, each relying on the difference in the subject’s properties, for example, size [15,16], mechanical properties [17,18], electrical properties [19,20], and magnetic response [21].

Electrokinetic (*EK*) manipulation, an active approach that uses the electric field as the external force, involves phenomena that stem from the electrical double layer (EDL), specifically electroosmotic flow (EOF) and electrophoresis (*EP*) [22]. EOF-pumping has demonstrated utility for submicron-scale manipulation [23]. Moreover, *EK* manipulation of fluid/particles has been used in the agri-food industry and for potable liquid purification [24,25,26,27]. Ion concentration polarization (ICP) is an *EK* phenomenon used for ion extraction [9] that results from coupled mass and charge transport via ion-permselective micro-nanofluidic interfaces [28]. The polarization of ion concentrations happens in nano-junctions upon applying voltages to the ends of the two nano-linked microchannels [6,29]. Due to the EDLs having dimensions in the order of the nano-junctions, they overlap and form an ion depletion zone (IDZ) on one end and an ion enrichment zone (IEZ) on the other. On the IDZ side, a pair of vortices emerge because of the dominant EDLs induced by the electrostatic forces on the electrolyte’s components, repelling/passing co-/counter-ions [30]. The discovery of the IDZ and IEZ phenomenon was observed using two U-shaped channels connected through 60-nm deep nano-junctions [31]. As an alternative to difficult-to-fabricate nano-junctions, a porous medium can also be used to connect these zones [32,33].

ICP has also been used for biomolecule preconcentration of proteins [34,35,36,37,38,39,40], DNA [41,42,43], viruses [44,45], and cancer cells [46]. In 2013, Jeon et al. [47] proposed continuous ICP particle separation with sizes ranging from 50 nm to 10 μm, with separation based on the lateral repulsive force arising from the 3D depletion zone. They reported that a key factor for this activity is the particle zeta potential ($\zeta_{p}$), rather than its size alone. As a result, it was found that high resolution deflection is achieved with zeta potential ratios on the order of ~2. In another study, Yoon et al. [48] investigated the forces acting on influenza A virus in an ICP-based manipulation device. They observed three phases of particle movement based on the balance between fluid drag and electrically-induced forces. They subsequently demonstrated the device’s capability to successfully focus 1 µm and 1 nm particles in a narrow stream [49].

Computational methods, alongside empirical observations, have yielded important insights where the device parameter space can readily be explored to optimize desired outputs. For instance, Daiguji et al. [50,51] developed a model for 2D-ICP simulation in 2004 by solving the flow field, ion transport, and electric field in an interactive way inside a nanochannel, demonstrating the relative impacts and importance of these factors. Jin et al. [52] further extended Daiguji’s model, where the transient behavior across an entire ICP device was investigated for the first time. Since then, several geometries/designs have been numerically investigated, including channels designed with X-shaped [53,54], U-shaped [55], single-channel [56,57], double-sided nano-interfaces [58,59,60], 3D-bifurcation [61], nanochannels edges [62,63], and parallel microporous membranes [64].

In addition to fundamental studies, the ICP concept has also been numerically explored for a wide range of applications. Squires et al. [65] conducted numerical examinations of a single channel’s parameters to develop an intuition for qualitative behavior and scaling relations for quantitative understanding, aiding the design of biosensors. Molecular concentration was investigated by Ouyang et al. [66] via solving the flow field and demonstrating scaling relations between system parameters. Further, Wei et al. [67] analyzed a multiwell-based ICP pre-concentrator device for matrix metallopeptidase 9 enzyme (from breast cancer) detection and reaction enhancement. Gong et al. [68,69] numerically modeled a double-sided channel and studied the operational and structural parameters for high-concentration Li^+^ extraction. ICP-based devices have also been used for efficient micro-scale fluid mixing induced by the ICP vortices [70,71], albeit not in the context of continuous separation activities.

Despite these various fundamental and applied studies, the investigation of ICP-based separation and isolation of submicron- or nano-scale particles/analytes is still limited in terms of understanding the impact of fluid flow on separation mechanisms. This work reports numerical insights into submicron-scale particle separation and isolation derived by ICP, where this can be controlled by changing the flow rate and applied voltages. First, a 2D-finite element model (FEM) was developed to validate *EK* phenomena comprising the coupled physics of electric field, fluid flow, and ion transport. Thereafter, the reciprocal effect of flow rates and applied voltages and their combinational impact on circulating ICP-induced vortices are examined. As a result, device functionality and performance can be assessed by the dimensionless parameters we introduce. We further investigate and compare two particle tracing models, whereafter we utilize one of these to study particle responses. Consequently, we describe four particle modes determining its final state as a function of its size and material makeup. These modes uncover whether the particles are trapped in or pass around the ICP-induced vortex.

## 2. Device Principle and Numerical Model

**Figure 1:**
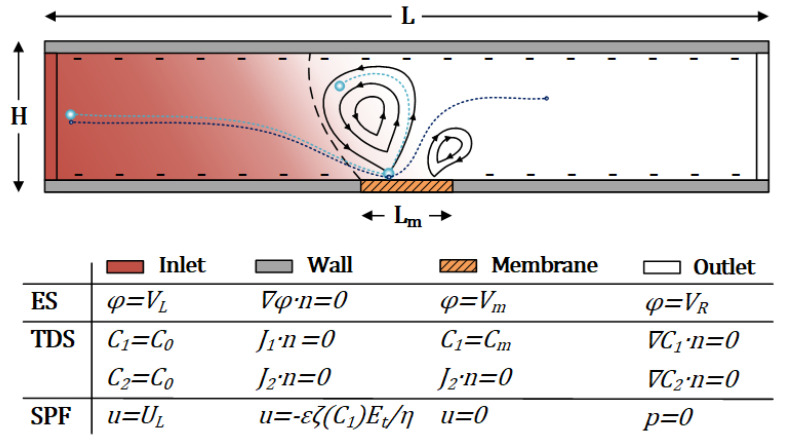
Schematic illustration of the numerical model and the boundary conditions associated with each set of physics. The nano-junctions are modeled as a constant concentration and voltage boundary condition. Larger particles (cyan) are trapped due to larger net forces, while smaller ones (blue) escape.

The interactive physics of the ICP phenomenon include electrostatics (ES), transport of diluted species (TDS), and single-phase flow (SPF), which are coupled in the numerical model. Figure 1 shows the schematic view of the ICP-based device with a membrane located in the middle along the bottom of the simulation domain. The channel dimensions are given in Table 1, alongside relevant physical properties. The voltage difference applied to the two ends of the microchannel causes EOF, where the membrane voltage generates a vortex pair owing to the differential ionic concentrations at the membrane edges. In this numerical model, the influence of the membrane is assumed as a boundary condition with a given concentration and an equivalent voltage across its length $L_{m}$ given the small scale of the membrane elements (e.g., nano-junctions) compared to the channel dimensions.

### 2.1. Electric Field

The electric field is governed by the Poisson’s equation and is solved in ES for the distribution of electric potential, φ, with:

(1) $$-\nabla \cdot \left(\varepsilon \nabla \varphi \right)=\rho_{e}=F\overset{n}{\underset{i=1}{\sum }}Z_{i}C_{i}$$

where the external space charge density ($\rho_{e}$) due to the existence of the charged ions with $C_{i}$ concentration and $Z_{i}$ valance, influences the electric field E=−∇φ within the electrolyte. Here, F is the Faraday’s constant, n is the number of ions, and the saline permittivity is given by [72]:

(2) $$\begin{array}{c} \varepsilon =\varepsilon_{w}\varepsilon_{0}(1-3.742\times 10^{-4}Tc+0.034c^{2}-0.178c+1.515\times 10^{-4}T\\ -4.929\times 10^{-6}T^{2}) \end{array}$$

which is a function of water’s relative permittivity ($\varepsilon_{w}$) and permittivity of free space ($\varepsilon_{0}$). Equation (2), thus, correlates with temperature, T [°C], and mean ionic concentration, $c=\frac{1}{n}\overset{n}{\underset{i=1}{\sum }}C_{i}$. Boundary conditions of the electric field are constant voltages at the inlet ($V_{L}$), outlet ($V_{R}$), and membrane ($V_{m}$). Wall boundaries are set to electric insulation (n·E=0, with n being the surfaces’ normal vectors) where the tangential electric field ($E_{t}$) drives the ions captured in EDL (EOF).

### 2.2. Concentration Field

Ion transport is governed by the Nernst-Planck equation in TDS, with:

(3) $$\frac{\partial C_{i}}{\partial t}=-\nabla \cdot J_{i}$$

(4) $$J_{i}=-\left(D_{i}\nabla C_{i}\pm \mu_{i}C_{i}\nabla \phi \right)+uC_{i},$$

where $C_{i}$ and $J_{i}$ are the concentrations and flux densities of the ions, respectively. In Equation (4), the contributions of diffusion, the electrostatic response of the ions to the local electric field, and convection are considered on its right-hand side. Here $\mu_{i}=Z_{i}\frac{D_{i}e}{k_{b}T}$ is the electrical mobility of the ions, wherein $k_{b}$ is the Boltzmann constant, and e is the elementary charge. Besides the interrelated terms in Poisson and Nernst-Planck equations, the fluid velocity field (u) in the convection term affects the migration of the ions. On the other hand, the mutual effect of the *EK*-driven flow is taken into account in the fluid flow physics, described in the next section. Here, the saline concentration in the reservoirs and the main channel in the initial case are $C_{0}$ (for both Na^+^ and Cl^−^), and distributed homogeneously. At the membrane, $C=C_{m}$ is applied for cations since the zeta potential of nano-junctions is negative. Thus, only the flux of anions is set to zero, as is the case with the impermeability condition applied to the walls.

### 2.3. Flow Field

The governing equations for the fluid flow in SPF are the continuity and Navier-Stokes equations. Considering incompressible, isothermal, laminar flow in steady-state, the equations are simplified to:

(5) ∇⋅u=0

(6) $$\rho \left(c\right)\left(u\cdot \nabla \right)u=-\nabla p+\eta \nabla \cdot \nabla u+\rho_{e}E-\frac{1}{2}E^{2}\nabla \varepsilon -\rho \left(c\right)g$$

where $\rho \left(c\right)=\rho_{0}+\beta c$, with linear density changes with solution concentration [73], p is the pressure field and g is the gravitational acceleration vector. The source terms on the right-hand side of momentum Equation (6) are Coulomb force (electrostatic force due to the net charge), dielectric force (due to the dielectric permittivity gradient), and buoyancy (produced from the density variations), respectively. These interplaying forces can cause electrohydrodynamic flow instabilities, albeit only when the electric field exceeds 100 V/cm [72], which is beyond the range of voltages used in the current study. Therefore, the last two terms in Equation (6) are ignored. The saline flows into the microchannel from the inlet with a constant velocity ($U_{L}$) and exits from the outlet with an atmospheric pressure boundary condition. The membrane is modeled as a no-slip boundary, and an electroosmotic slip velocity was assigned to the negatively charged walls of the channel, with:

(7) $$u_{EOF}=-\varepsilon \zeta \left(C_{1}\right)E_{t}/\eta$$

where $\zeta \left(C_{1}\right)=20\;\log_{10}\left(C_{1}\right)$ [mV] is the wall zeta potential for concentration < 1 [M] [72].

### 2.4. Particle Tracing Approaches

We investigate two particle tracing approaches, Newtonian and massless, to determine the most physically realistic and computationally efficient method for particle studies.

#### 2.4.1. Newtonian

Since the particles’ size determines whether they are trapped in or pass around the ICP-induced vortex, they are modeled as modified Lagrangian points, including their interactions with walls. For realistic particle tracing, Newton’s second law of motion is used, with:

(8) $$m_{p}\frac{\partial U_{p}}{\partial t}=F_{D}+F_{EK}+F_{W}$$

where $F_{D}$, $F_{EK}$, and $F_{W}$ are the forces due to hydrodynamic drag, *EK*, and wall bounce, respectively. The drag force is related to the difference between the particle’s velocity ($U_{p}$) and its surrounding fluid’s ($u\left(u,v\right)$). Using Stokes drag gives:

(9) $$F_{D}=\frac{m_{p}}{\tau_{p}}\left(u-U_{p}\right),$$

where $m_{p}$ is the mass of a perfect sphere particle with a diameter of $d_{p}$ and density of $\rho_{p}$. Additionally, $\tau_{p}=\frac{\rho_{p}d_{p}^{2}}{18\mu }$ is the particle velocity response time.

The *EK* force is the summation of the *EP* and dielectrophoretic (DEP) forces, written as:

(10) $$F_{EK}=F_{EP}+F_{DEP}=3\pi \zeta_{p}\varepsilon d_{p}E+\frac{1}{2}\pi \varepsilon d_{p}^{3}f_{cm}\left(E\cdot \nabla \right)E,$$

where $\zeta_{p}$ is the particle’s zeta potential and $f_{cm}$ is Clausius-Mossotti factor. The first term determines the particle *EP* and the latter describes the particle’s tendency to migrate to the weak/intense electric field, known as nDEP/pDEP. Usually, these two forces have negative values for bioparticles. To permit appropriate particle-wall interactions, the particle experiences a repulsive force $F_{W}$ from the wall to avoid particle-wall overlapping.

#### 2.4.2. Massless

*EK*-based devices work at a relatively low Reynolds number (Re) [74]. Therefore, particle inertia can be mostly neglected, meaning that the particle reaches the relaxation state in an infinitesimal time scale [75]. At that point, the forces balance, and the explicit formulation yields:

(11) $$U_{p}=u+u_{EK}=u+\frac{F_{EP}}{f}+\frac{F_{DEP}}{f},$$

where $f=\frac{m_{p}}{\tau_{p}}=3\pi \eta d_{p}$ is the Stokes frictional factor for a spherical particle in a creeping flow. Using this method, in most *EK*-driven cases, the particle path can be quickly estimated at the cost of negligible deviation from its exact trajectory. Same as in the previous model, the particle-wall interaction is taken into account using the reflection velocity condition.

### 2.5. Computational Implementation

To solve the above-mentioned equations, a 2D FEM-based model is developed in COMSOL Multiphysics 5.3 (Burlington, MA, USA) to emulate the ICP phenomenon in a rectangular domain. In addition, the particle tracing for the fluid flow module was used to simulate the particles’ motion. The 2D simplification has been utilized elsewhere [60] as ICP effects predominantly occur along the channel width, given that the ICP membrane is typically the same height as the channel, without any out-of-plane effects, which would only be relevant in alternative channel/membrane geometries with non-uniform z-direction cross sections [76]. The interconnection between the physics is introduced through body forces and source terms in each equation. The structured mesh was generated with refinement around the membrane and walls to capture adverse velocity and/or concentration gradients. Nonlinear elements were used for the sake of accuracy, and the equations were solved by multi-frontal massively parallel sparse and automatic Newtonian damping factor selection.

## 3. Result and Discussion

### 3.1. Method Validation

**Figure 2:**
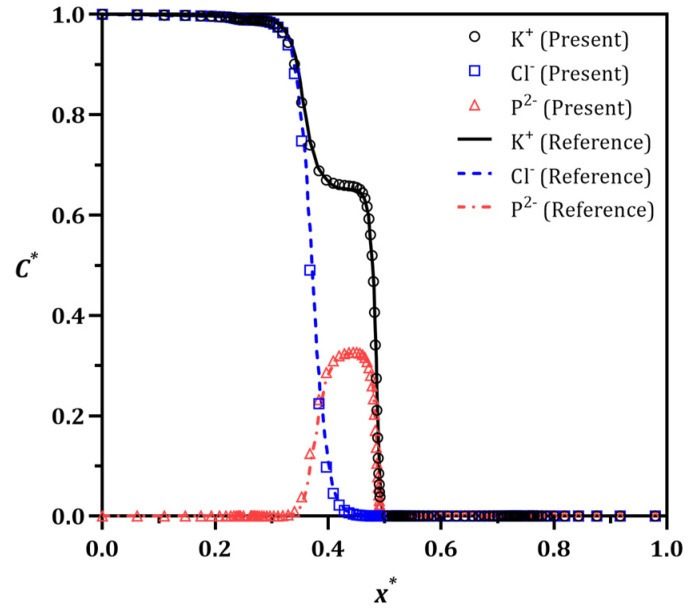
Validation study. The distribution of the ions concentration modeled in the present study and that of reported by Liu et al. are compared [60] after the formation of IDZ at $V^{*}=1.7$. The concentration of the charged species drops before the membrane located in the middle of the channel length.

Figure 2 depicts the comparison between the present method and that of reported by Liu et al. [60], based on the dimensionless concentration ($C^{*}=\frac{C}{C_{0}}$) of K^+^, Cl^−^, and P^2−^ along the centerline of the channel ($x^{*}=\frac{x}{L}$). There is a good agreement between the present results and their numerical work carried out by COMSOL Multiphysics. The distribution of the charged species drops around the midpoint of the channel (near the membrane), after which it almost vanishes. We further show that the time-dependent flow vortex behavior in an experimental system analogous to that simulated here is similar as well, as shown in Video S1 (top video is from [33], bottom of video is simulated flow field and ion distribution from the present work) in Electronic Supplementary Information (ESI). The present model was further validated as illustrated in Figure S1 in ESI. Here, the $V_{m}$ is altered to fine-tune the cross-membrane voltage, $V_{cm}=\frac{V_{L}+V_{R}}{2}-V_{m}$, leading to a non-dimensional voltage, $V^{*}=\frac{V_{cm}}{V_{L}}$, of 1.7. The higher $V^{*}$ causes more intense vortices, hence larger IDZ.

### 3.2. Mesh Sensitivity Analysis

**Figure 3:**
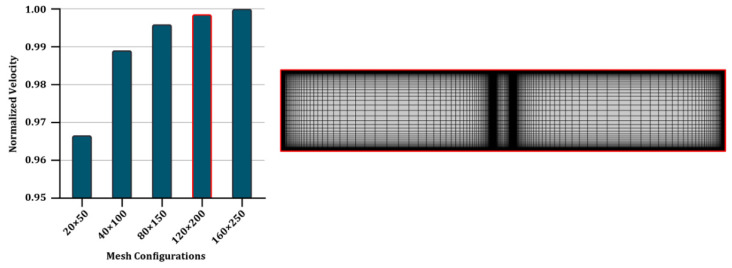
Mesh sensitivity analysis and the whole meshed domain using the final grid configuration (120 × 200), with highest mesh density in the vicinity of channel walls and in the ICP vortex domain. The red border of the fourth column indicates the selected mesh configuration.

In order to analyze the sensitivity of the solution to the mesh, five different mesh configurations were utilized in Figure 3. The numbers indicate the mesh counts over the membrane domain along the x and y directions, respectively. As shown from the charts, the trend of the normalized velocity sampled close to the top of the membrane converges gradually. Therefore, compared to the finest grid, a 120 × 200 case with an overall ~72,000 nodes (the whole meshed domain is also shown at the right) is considered to benefit from both the solution accuracy (error < 0.2%) and the calculation time for the rest of the study.

### 3.3. The Effect of Voltage/Flow Rate on IDZ

The mechanism of vortex generation is first studied to evaluate the device performance for size-based particle separation. The generation of ICP vortices in a continuous flow system can be significantly affected by the two key parameters, $V^{*}$ and the flow rate. As a result, efficient particle separation can be achieved when the right combination of these is chosen.

**Figure 4:**
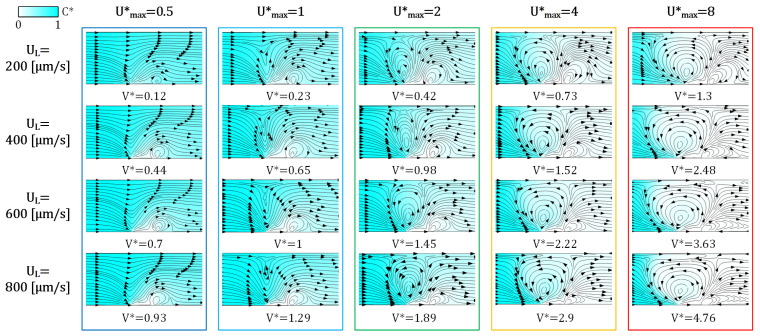
The influence of $U_{L}$ and $V^{*}$ is categorized by $U_{max}^{*}$ for measuring the vortex size and ICP performance. Arrows demonstrate the fluid flow direction. The critical point, $U_{max}^{*}=1$, is where the main ICP vortex is produced from the central stagnation point for the first time. By increasing $U_{max}^{*}$, another stagnation point forms over the vortex, and an IDZ develops.

To do so, the vortex velocity (or ICP velocity, $U_{ICP}$) can be scaled by the lateral flow velocity ($U_{L}$), the flow rate divided by the channel cross-sectional area. Importantly, the exact $U_{ICP}$ cannot be readily directly calculated in the absence of a numerical model, especially due to the combined effect of EOF and ICP secondary flow. To isolate the impact of *EK* effects, the $U_{L}$ is excluded from the calculation of the total fluid velocity; hence, the overall calculated (*EK*) velocity is assumed to be solely caused by the ICP phenomenon. This simplification is appropriate as the summation of these effects impacts the vortex shape and size. Henceforth, the vortex intensity depends on the maximum of the new dimensionless velocity that is defined as:

(12) $$U^{*}=\frac{U_{ICP}}{U_{L}}=\frac{U_{L}-u}{U_{L}}=1-\frac{u}{U_{L}},$$

where the large values of $U^{*}$ correspond to the dominance of the ICP flow as shown in Figure 4. To ensure equivalent vortex formation at different inlet flow velocities, $V^{*}$ is adjusted accordingly. Importantly, this scaling allows us to characterize the flow field regardless of the specific combination of $U_{L}$ and $V^{*}$. As expected, at high inflow velocities, higher values of $V^{*}$ are required to obtain an equivalent flow pattern. In $U_{max}^{*}<1$, only IDZ small side-vortex emerges, leaving all species to pass through. While at $U_{max}^{*}=1$, the second vortex appears near the center of the channel over the membrane’s corner. This condition is the critical point for the IDZ occurrence, namely the development of the stagnation point at the vortex’s center. For $U_{max}^{*}>1$, the main vortex has already formed and the ICP starts to push the ions back towards the channel entrance. At the beginning of the transition, the right-side vortex starts to grow until the left one expands and squeezes it. As a consequence, streamlines/pathways of the passing flow are narrowed, leading to a smaller gap for particles to escape under the vortex, with $U_{max}^{*}≳2$ resulting in the vortex extent reaching the opposite side of the channel in Figure 4, and thus all passing particles being subject to vortical capture above a given cut-off size. Moreover, at a larger $U_{max}^{*}$ the vortex growth stops height-wise and gradually continues lengthwise. The input flow can thus be a useful tool to control the vortex size, hence the particle cut-off size for trapping or passing the desired particles.

**Figure 5:**
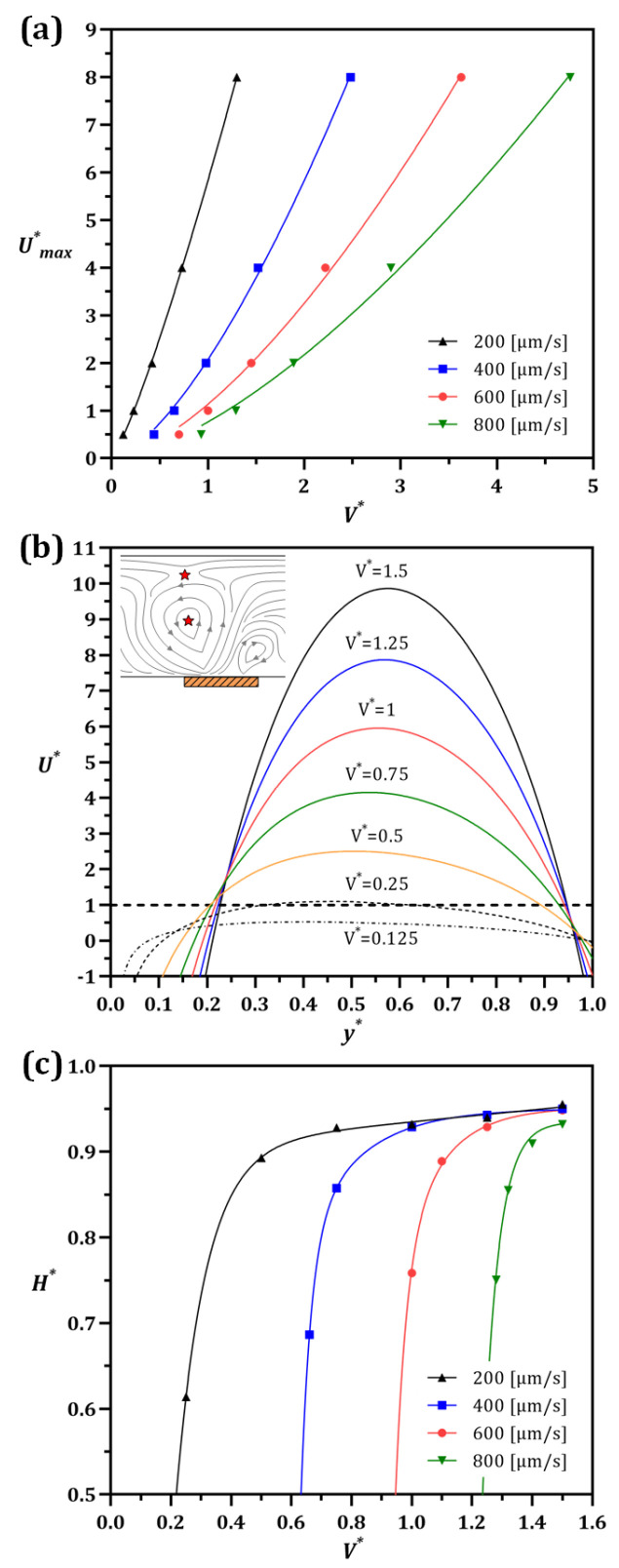
ICP adjustments. (**a**) The nonlinear relationship between $U_{max}^{*}$ and $V^{*}$ estimated and formulated by $U_{max}^{*}=a{V^{*}}^{n}$ at each lateral velocity. (**b**) The $U^{*}$ calculated along the channel height starting from the left corner of the membrane toward the channel roof at $U_{L}=200$ [µm/s]. The brown rectangle indicates the membrane’s location. The stagnation locations are highlighted by the red stars in the subset and the dashed line when $U^{*}=1$. If the interception happens twice, it means the vortex has already emerged. (**c**) The variation of the dimensionless vortex size ($H^{*}$) by the voltage difference ratio at different $U_{L}$.

Furthermore, at a certain $U_{max}^{*}$, higher $V^{*}$ are required to achieve similar flow/vortex patterns at higher $U_{L}$. The relationship between $U_{max}^{*}$ and $V^{*}$ at different $U_{L}$ values is estimated via power-law curve fitting ($U_{max}^{*}=a{V^{*}}^{n}$) with $R^{2}>99.7\%$ in Figure 5a. The full report of the regression is provided in Table S1. Considering the low voltage and flow requirements for the majority of lab-on-chip applications, in this figure an inlet velocity of $U_{L}<800$ [µm/s] was used. As a result, a significantly lower electric field (<100 [V/cm]) was required for efficient particle separation.

Figure 5b shows how $U^{*}$ changes along the channel height for different $V^{*}$ values when $U_{L}=200$ [µm/s]. The voltage difference amplifies the fluctuations in the flow velocity and increases the kurtosis of the $U^{*}$ curve, while lowering the $V^{*}$ reduces the ICP velocity and flattens the curve. As discussed earlier, once $U^{*}$ reaches unity, the stagnation point forms, after which the ICP vortex emerges with two stagnation points. Therefore, for those cases where the $U^{*}$ curve crosses unity twice, the two corresponding heights $y^{*}$ indicate the positions of the center and the tip of the vortex from left to right on the horizontal axis, respectively (Figure 5b). Moreover, the $U^{*}$ curve for the smaller $V^{*}$ has no interception with $U^{*}=1$, signifying that there is no vortex formed.

Figure 5c illustrates the extracted vortex sizes ($H^{*}=\frac{H_{vortex}}{H}$) via measuring the height of the top stagnation point ($H_{vortex}$), namely the streamline saddle point. For all the flow velocities and $V^{*}$ tested, it is shown that the vortex height increases until it reaches a threshold, quantified here as $H^{*}=95\%$. At this point, the vortex is fully developed and maximally fills the cross-sectional area of the channel. This condition is a key requirement to achieve an efficient submicron particle separation. Moreover, a vortex forms, and reaches its max height more readily at higher flow velocities, albeit with higher $V^{*}$.

### 3.4. Newtonian vs. Massless Particle Tracing Models

To further understand the separation mechanism via tracing different particles, a computationally cost-efficient and fast particle tracing model should be first selected. Here, we chose *E. coli* bacterium and a virus as our model particles because of their distinguishable size difference and biological relevance. *E. coli* bacterium ($\rho_{p}=1085$ [kg/m^3^]) and the virus ($\rho_{p}=1180$ [kg/m^3^]) are approximately 1 µm [77] and 150 nm [78] in diameter, respectively. Based on their different dimensions and densities, the virus is expected to pass through while the bacteria become trapped in the vortex. All particles were assumed to be electrically neutral to minimize the complexity at this step. To achieve a stable nanoparticle extraction from a mixed solution, the device’s working condition is $U_{L}=400$ [µm/s] (corresponding to $U^{*}\;\approx \;3$ in Figure 4) and $V^{*}=1.25$.

**Figure 6:**
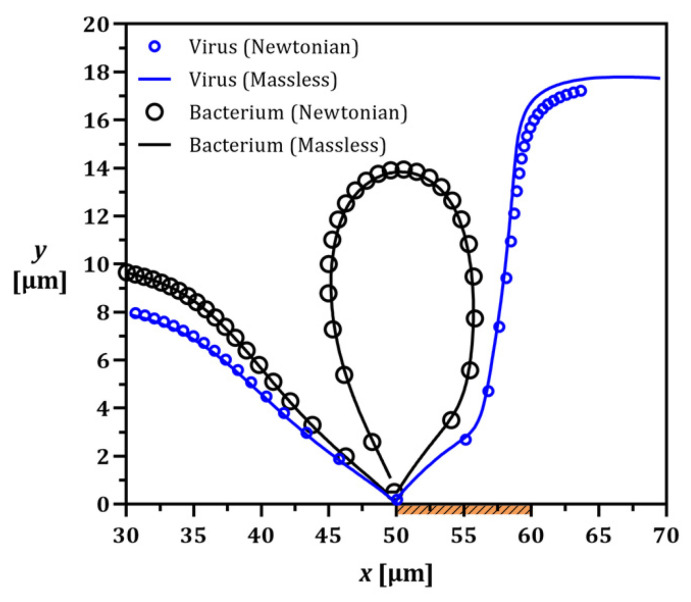
Comparing the trajectories of the electrically neutral bacteria and virus using modified Newtonian and massless models to enable the particle size and wall effects. The virus escapes beneath the vortex while the bacterium is forced to follow the vortex streamlines.

In the presence of an ICP-induced vortex (Figure 4), particles are directed toward the membrane following the fluid streamlines. Once reaching the membrane, the particle size determines whether it gets trapped within or escapes from the vortex. As shown in Figure 6, both particle tracing methods led to nearly the same results with minor discrepancies. However, the significant difference between the two is the computation time, where the massless method is more than three orders of magnitude faster with equivalent particle trajectories. Here, the massless method is faster as particles here effectively follow fluid streamlines compared to mass-containing particles whose position much be iteratively calculated as they inertially diverge from those streamlines. Hence, this model is used for calculating the critical particle size, which defines the boundary between the trapping and escaping modes.

### 3.5. Particle Separation Mechanism

**Figure 7:**
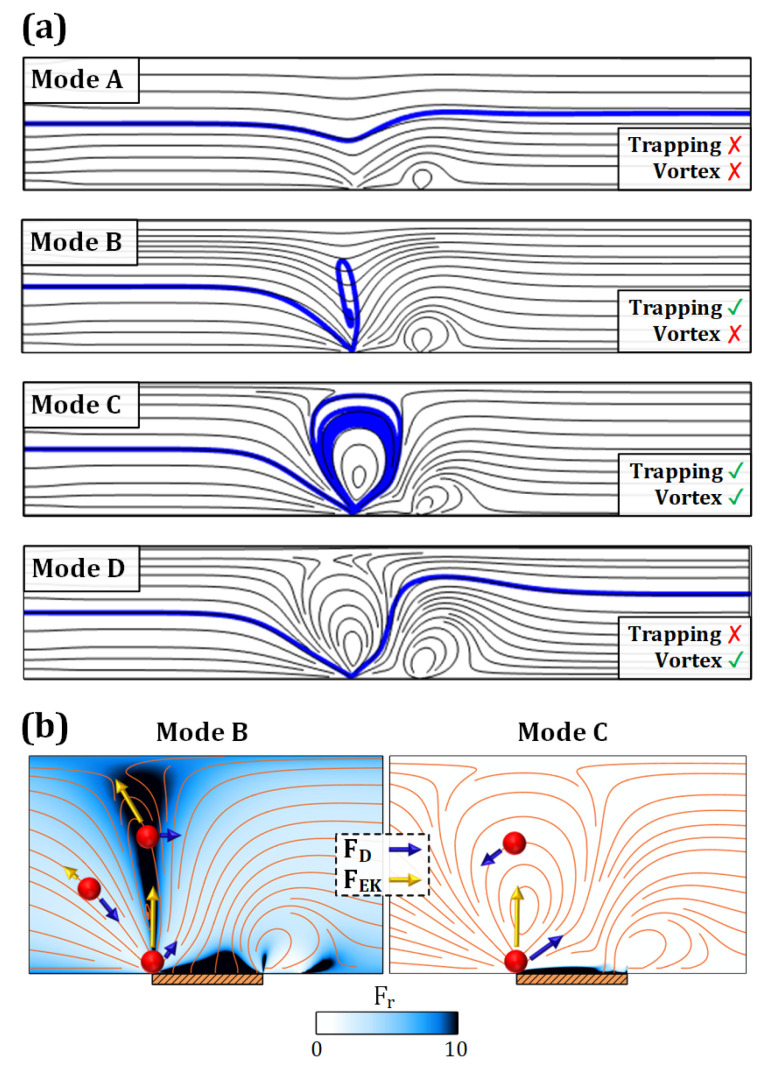
Particle trapping/escaping modes. (**a**) Different particle modes occurrence mainly because of particle *EP* (fluid streamlines are shown). Mode A (Allowed): no vortex and no trapping, Mode B (Blocked): no vortex but trapping, Mode C (Captured): fluid vortex and trapping, and Mode D (Dodged): escaping from the vortex. The last two modes are desirable for multi-particle separation, where both particle size and charge are decisive. (**b**) Diagram of forces acting on negatively charged particles at different locations during modes B and C (the streamlines are for particle velocity and not fluid velocity). As particles advance toward the membrane, the $F_{EK}$ rises due to the high electric field and its gradient. In mode B, *EK* force slows down the particle before reaching the membrane, and together with the drag force, induces a spiral-like motion to particles even without the existence of a vortex. This only happens for a particular condition where *EP* dominates the drag force at the high electric field area. It should be mentioned that DEP is only significant within a small bandwidth (1 [µm]) above the membrane. For instance, in mode C, the particle is captured by its size and DEP motion.

It has been observed that the effect of particle charge is significant in ICP-based particle manipulation [47]. Here, investigations also demonstrate that $\zeta_{p}$ plays a significant role in determining particle trajectories, which we define as occurring in one of four distinct modes. Figure 7a shows the particle modes that occur due to the contribution of $F_{EP}$. The first mode (mode A: Allowed) takes place if there is no vortex formed. In case of a large *EK* force, particles circulate within the *EK* vortex, where mode B (Blocked) begins as the force ratio $F_{r}=\left|\frac{F_{EK}}{F_{D}}\right|$ exceeds 1, where *EK* and drag forces act in opposition across most of the domain.

These forces are schematically illustrated in Figure 7b. The electric field is amplified around the depletion area owing to the presence of the membrane, which leads to the amplification of particle *EP* retardation encountering a blockade in the proximity of ICP-induced flow. Hence, similar to the ion depletion effect, *EP* force pushes particles and can be more crucial to particle destination than the net drag force. Therefore, in a case of high $F_{r}$, even a small particle can be trapped in the intensified electric field if $F_{EP}$ shifts the particle’s streamline. That is despite the fact that DEP also takes part in trapping modes B and C (Captured), albeit only for a short time around the membrane where high electric field gradients exist.

More importantly, mode C occurs with relatively larger particles, as discussed in the previous section. Therefore, particles below a critical threshold will occur in mode D (Dodged), undergoing mild $F_{EK}$. For multi-particle separation purposes, modes C and D are more desirable and can provide a stable differentiation of particles with distinct sizes. On that account, the critical zeta potential ($\zeta_{cr}$), defined as the zeta potential at which the transition between the modes occurs, is further studied for a wide range of operational parameters in the following section.

**Figure 8:**
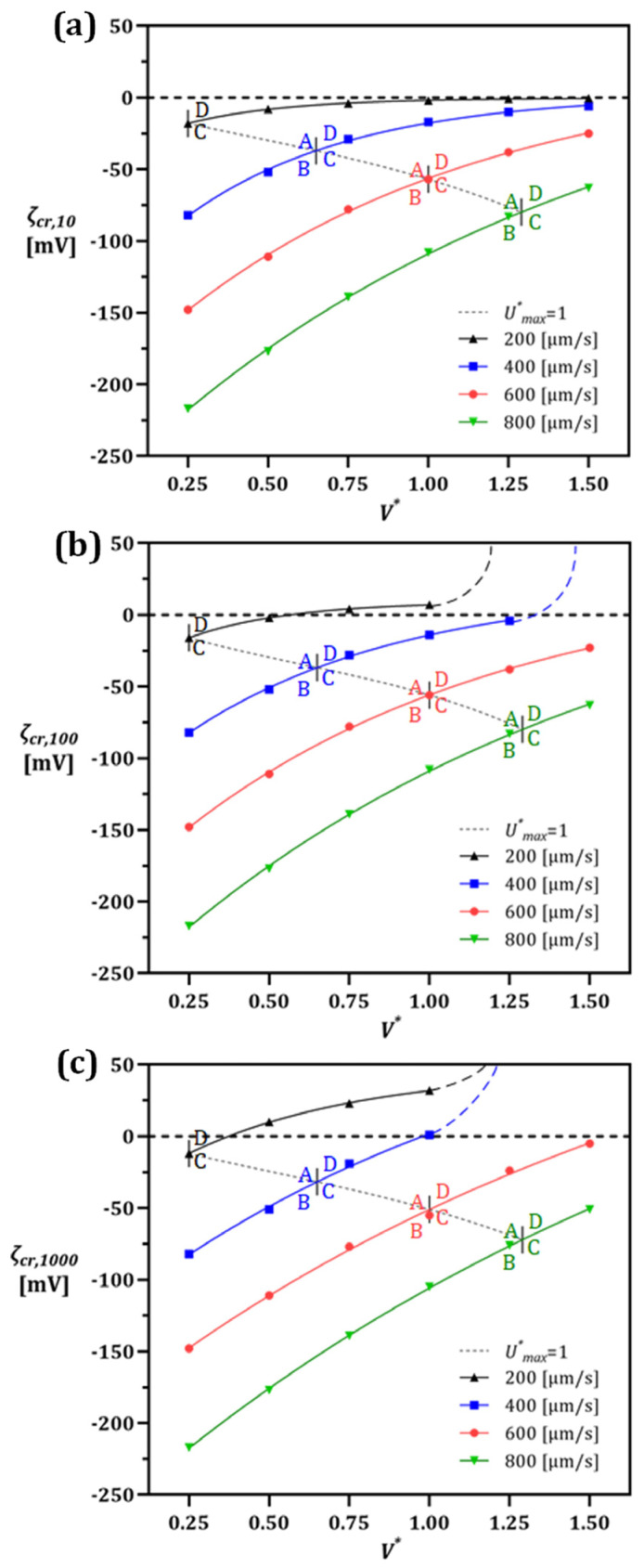
Critical $\zeta_{p}$ for submicron particle sizes of (**a**) 10 nm, (**b**) 100 nm, and (**c**) 1000 nm at different inlet velocities and $V^{*}$. The intersections of critical $\zeta_{cr}$ and $U_{max}^{*}$ lines divide the graphs into four regions at every flow rate, each representing a mode for the corresponding particle. As a result, particles with known charge and size can be separated by adjusting $U_{L}$ and $V^{*}$. Each letter demonstrates different modes, which introduced as A (Allowed), B (Blocked), C (Captured) and D (Dodged).

Investigating the effect of $V^{*}$ and $U_{L}$ in more detail, the parameter $\zeta_{cr}$, which also determines the particle manipulation mode, is plotted in Figure 8 for three particle sizes of 10, 100, and 1000 nm for $V^{*}=0.25–1.5$ and $U_{L}=200–800$ [µm/s]. The particles are modeled with constant charges, and the streamline deviation from a model with concentration-dependent $\zeta_{p}$ is neglected in the deionized domain. Next, a factorial test was performed to further explore the principle behind the trade-off between $\zeta_{cr}$ and its corresponding $d_{p}$. It is shown that for a particular particle ($\zeta_{p}$, $d_{p}$) at a specific inlet velocity, there is a critical $V^{*}$ at which the vortex starts to evolve. These points are marked on each $\zeta_{cr}$ line plotted for a given inlet velocity in Figure 8 and are connected with a dashed line along which $U^{*}=1$. The data presented in Figure 8 can be further understood using the following example. Considering a 100 nm particle and $U_{L}=600$ [µm/s] (Figure 8b), modes A/B occur only until the $\zeta_{cr}$ reaches approximately −55 mV, corresponding to the critical $V^{*}\;≅\;1$. However, above this critical $V^{*}$, where the vortex is already formed, only modes C/D take place. More specifically, once $V^{*}>1$, either C or D can occur if $\zeta_{p}$ is below or above the $\zeta_{cr}$ line, respectively, for that specific inlet velocity.

The positively charged particles are also included to determine the transition point where the vortex size is large enough that only leaves a narrow path on the order of a particle radius for the flow to pass through. This point is highlighted by the $\zeta_{cr}=0$ dashed line for neutral particle manipulation, where the particle is sufficiently large to be captured by the vortex.

Mode C dominantly occurs at higher $V^{*}$ due to the intensified vortex. Therefore, the trends are followed by a dashed line indicating that even unrealistic positive values of $\zeta_{p}$ cannot prevent the particle from escaping the vortex.

Given the magnitude analysis of the forces, the mode shift criterion is explained quantitatively by conducting a factorial test over the influential parameters. Therefore, as shown in Figure 8, the value of $\zeta_{cr}$ was investigated within the device’s operational condition i.e., $V^{*}$ (0.25–1.5) and $U_{L}$ (200–800 [µm/s]) for different sizes of the particles $d_{p}$ (10, 100, and 1000 nm). The particles were modeled with constant charges, and the streamline deviation from a model with concentration-dependent $\zeta_{p}$ is neglected in the deionized domain. It is shown that for any particle size, regardless of the device condition, there is a threshold for $\zeta_{p}$ above/below which the particle escapes/traps. Moreover, the corresponding critical $V^{*}$ at which the vortex starts to evolve are marked on $\zeta_{cr}$ lines at any given flow rates. Knowing the calculated $U^{*}=1$ condition (dashed line), the vortex existence hence the actual mode is determined.

The data presented in Figure 8 may be further explained using the following example. Considering a 100 nm particle and $U_{L}=600$ [µm/s] (Figure 8b), Modes A/B occur only until the $\zeta_{cr}$ reaches approximately −55 mV, corresponding to the critical $V^{*}≅1$. However, above this critical $V^{*}$, where the vortex is already formed, only Modes C/D take place. More specifically, once $V^{*}>1$, either C or D can occur if $\zeta_{p}$ is below or above the $\zeta_{cr}$ line, respectively, for that specific inlet velocity. This example is also true for positively charged particles and the $\zeta_{cr}=0$ condition for neutral particle manipulation. In the latter case, a particle can be captured only due to the vortex drag force as a result of its radius being larger than the gap under the vortex (i.e., size-based separation).

After finding the interaction between a particle and its surrounding domain for these discrete cases, a regression function is applied (detail in Table S2), using which these results can be generalized in determining analytical relationships. In doing so one can readily estimate the value of $\zeta_{cr}$ by providing an initial $\zeta_{p}$, particle size, inlet velocity $U_{L}$, and $V^{*}$.

A regression function in the form of $\zeta_{cr}\left(d_{p},U_{L},V^{*}\right)=a-\left(a-b\right)e^{-cV^{*}}$ is utilized here. The parameters a, b, and c are primarily a function of $U_{L}$ and $d_{p}$. As a result, the following expressions were extracted (coefficient of determination $R^{2}\;≅\;1$), with

(13) $$\begin{array}{c} \;a=3.75-0.0439U_{L}+1.28\times 10^{-4}U_{L}^{2}+0.0208d_{p}+4.16\times 10^{-5}d_{p}U_{L}\\ +1.4246\times 10^{-7}d_{p}U_{L}^{2},\\ \;b=-4.158-0.297U_{L}-3.707\times 10^{-5}U_{L}^{2},\\ \;c=\frac{1016.1+1357.45e^{-0.001094d_{p}}}{{\left(U_{L}-46.609\right)}^{1.231}} \end{array}$$

**Figure 9:**
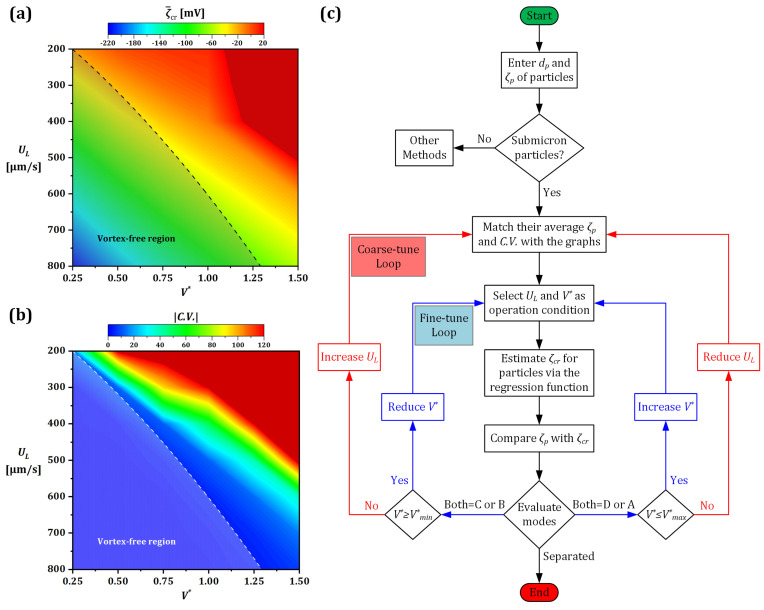
An algorithm for the separation of two particles of different sizes and charges. (**a**) The contours of the average critical zeta potential and (**b**) its absolute coefficient of variation for submicron particles. The scarlet region approximately with $U_{L}<500$ [µm/s] and $V^{*}>1.1$ is unpredictable due to the intensive vortex emergence that draws the particles inside; therefore, these working points are excluded from the device configuration in this study. (**c**) Flowchart/algorithm developed to separate two different particles with distinct sizes and charges.

In order to systematically define the operating conditions for separation, Figure 9 presents the contour plots of the key dimensional operational parameters ($U_{L}$ and $V^{*}$) and an algorithm developed for optimal particle separation. The average values of $\zeta_{cr}$ and its coefficient of variation (C.V.) are illustrated in Figure 9a,b. These values were produced using simulations run for a wide range of particle sizes 10–1000 nm and were analyzed using the proposed regression function. As a key result, the vortex-free region was determined since it enables the selection of initial values for the key parameters, which helps to estimate the presence of the four modes more precisely. Moreover, the critical zeta potential varies smoothly in the vortex-free region, however, as shown in Figure 9b, its variation is more radical once the vortex forms, especially at lower inlet velocity and higher $V^{*}$ region.

The flowchart/algorithm demonstrated in Figure 9c provides a useful tool to readily identify the key parameters required for the separation of two distinct submicron scale particles. The separation is met once the condition of having two different modes for two particles, i.e., one particle in trapping mode (B or C) and the other in escaping mode (A or D), is satisfied. The coarse- and fine-tuning parameters here are $U_{L}$ and $V^{*}$, respectively.

## 4. Conclusions

The presence of ICP-induced vortexes is a phenomenon that can be used for particle separation and capture, though has not previously been the subject of in-depth investigation. In the present study, a 2D-FEM numerical model is used to capture the multiphysical effects relevant to achieving submicron particle separation. Vortex formation is evaluated using dimensionless parameters indicating the voltage and velocity intensities. Accordingly, the correlation between the flow characteristics, inflow (200–800 [µm/s]), and applied voltages (0–2.5 [V]) are established and examined in detail. A scaling-based examination of the system permits the extent of the vortex to be quantified as a flow velocity ratio, where a dimensionless velocity of $U^{*\;}=1$ can be used to indicate the presence of an ICP-induced vortex. Further, four ICP flow modes (here given as A, B, C and D) have been identified using a massless particle tracing approach, where these modes denote whether particles are trapped in or escape from either of the two ICP-induced vortexes on either side of the nano-junctions; this massless method yields equivalent results as Newtonian particle trajectories, but is solved for three orders of magnitude faster. It was found that the difference in the charges of the particles is especially decisive in determining particle behavior, where high electric field gradients occur in the proximity of the membrane and impact particles in its vicinity. The critical zeta potential ($\zeta_{cr}$) is calculated for 10, 100 and 1000 nm particles to extract a performance regression function for device tuning. Moreover, the key functional parameters of the device were analyzed using an algorithm that provides a functional tool to readily determine the key parameters required to separate two different submicron-sized particle populations. These findings further reveal the promise of ICP flow-enhanced separation, not only for implementation in ionic separation activities as previously shown, but also for capture and separation and/or isolation of submicron particles and specimens. These findings contribute to a better understanding of ICP-based particle separation and isolation, as well as laying the foundations for the rational design and optimization of ICP-based sorting systems for a variety of applications.

## Figures and Tables

**Table 1:** Model parameters.

Parameter	Value	Unit	Description
L	110	[µm]	Length of the microchannel
$L_{m}$	10	[µm]	Length of the membrane
H	20	[µm]	Height of the microchannel
$V_{L}$	0.5	[V]	Left reservoir voltage
$V_{R}$	0	[V]	Right reservoir voltage
$V_{cm}$	0–2.5	[V]	Cross-membrane voltage
$U_{L}$	200–800	[µm/s]	Inlet velocity
$T_{0}$	300	[K]	Reference temperature
$\rho_{0}$	1000	[kg/m^3^]	Fluid density
η	0.001	[Pa·s]	Fluid dynamic viscosity
$D_{1}$	1.34 × 10^−9^	[m^2^/s]	Diffusion coefficient of cation
$D_{2}$	2.03 × 10^−9^	[m^2^/s]	Diffusion coefficient of anion
$Z_{1}$	+1	[-]	Cation (Na^+^) covalence
$Z_{2}$	−1	[-]	Anion (Cl^−^) covalence
$C_{0}$	1	[mM]	Bulk concentration
$C_{m}$	2	[mM]	Membrane concentration
β	40.908	[g/mol]	Expansion coefficient
$\varepsilon_{w}$	78	[-]	Bulk relative permittivity

## Data Availability

Not applicable.

## Supplementary Materials

The following supporting information can be downloaded at: https://www.mdpi.com/article/10.3390/mi13122203/s1, Figure S1: The validation of the present 2D-FEM based on the ICP modeling in a U-shaped channel at V = 1 [V] and P_0_ = 0 [Pa] [55]. (a) Anion concentration distribution and flow directions inside the channel and nanochannels. (b) Electric potential and its field intensity along the channel centerline. (c) Ion concentration and pressure changes along the centerline of the U-channel. The current numerical scenario shows a strong performance in predicting alterations in every solution variable. Table S1: The regression results for $U_{max}^{*}=a{V^{*}}^{n}$; Table S2: The regression results for $\zeta_{p}=a-\left(a-b\right)exp\left(-cV^{*}\right)$; Video S1: Time-dependent flow vortex behavior validation as a proof-of-concept. An experimental system in the top video is taken from [33] and the current simulation is shown in the bottom of video. The simulation and the observation are in good agreement.

## Abbreviations

Dielectrophoresis (DEP); Electrical double layer (EDL); Electrokinetic (*EK*); Electroosmotic flow (EOF); Electrophoresis (*EP*); Finite element method (FEM); Ion concentration polarization (ICP); Ion depletion zone (IDZ); Ion enrichment zone (IEZ).
