# Supplementary material for: Continuous Submicron Particle Separation Via Vortex-Enhanced Ionic Concentration Polarization: A Numerical Investigation
Source: Micromachines (Basel). 2022 Dec 12;13(12):2203. doi: 10.3390/mi13122203 (PMC9786152; doi:10.3390/mi13122203)
Supplement: Supplementary file 1 [file micromachines-13-02203-s001.zip › micromachines-2007779-supplementary.pdf]

micromachines

MDPI

Supplementary Information

Continuous Submicron Particle Separation via Vortex-Enhanced Ionic Concentration Polarization: A Numerical Investigation

Rasool Dezhkam [1,2,3,†], Hoseyn A. Amiri [1,2,†], David J. Collins [4,5,*] and Morteza Miansari [1,2,*]

1 Micro+Nanosystems and Applied Biophysics Laboratory, Department of Mechanical Engineering, Babol Noshirvani University of Technology, Babol 4714873113, Iran
2 Department of Cancer Medicine, Cell Science Research Center, Royan Institute for Stem Cell Biology and Technology, ACECR, Isar 11, Babol 4713818983, Iran
3 Department of Mechanical Engineering, Sharif University of Technology, Tehran 113658639, Iran
4 Department of Biomedical Engineering, University of Melbourne, Melbourne, VIC 3010, Australia
5 The Graeme Clark Institute, The University of Melbourne, Melbourne, VIC 3010, Australia
* Correspondence: david.collins@unimelb.edu.au (D.J.C.); morteza.miansari@synthego.com (M.M.)
† These authors contributed equally to this work.

1. Validation of U-Shaped Channel

To ensure the accuracy of the current 2D model, its performance has been further investigated for a U-shaped ICP channel [1]. Figure S1 shows the comparison between the model used in the present work and the results of the reference paper. Every influential parameter follows the reference trend precisely.

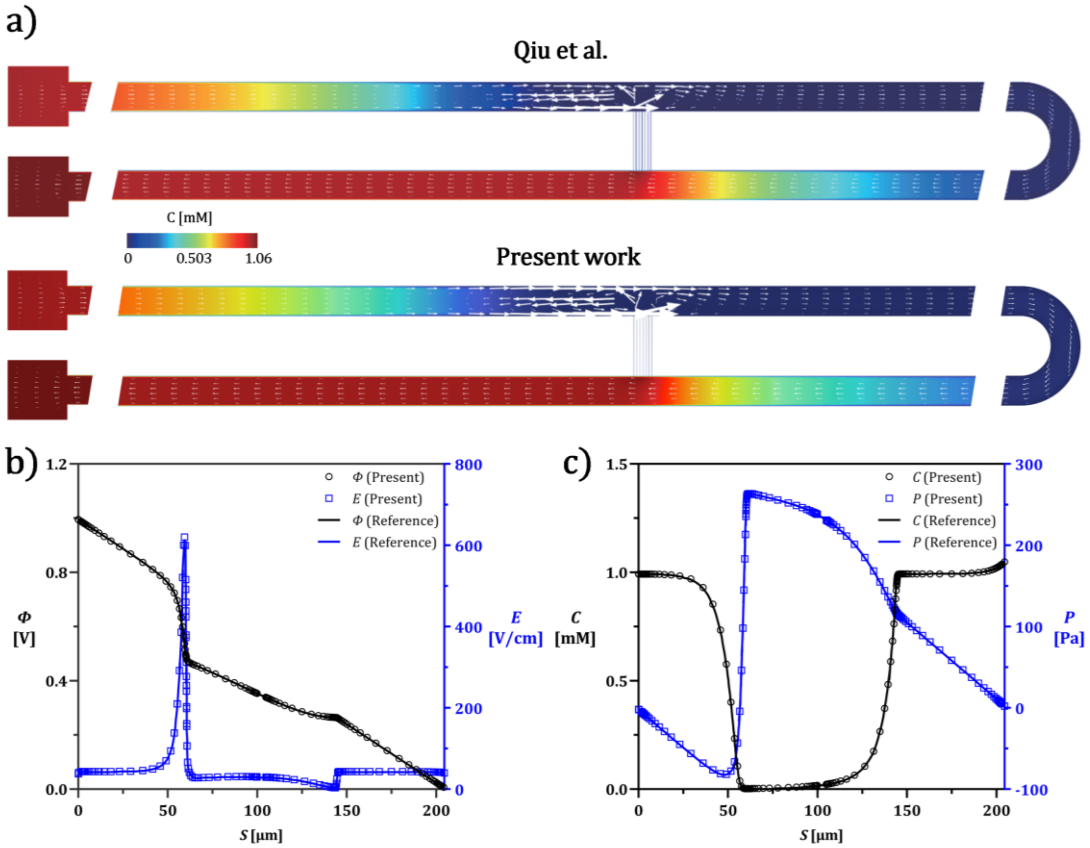

Figure S1. The validation of the present 2D-FEM based on the ICP modeling in a U-shaped channel at V=1 [V] and P_0=0 [Pa] [1]. (**a**) Anion concentration distribution and flow directions inside the

channel and nanochannels. (**b**) Electric potential and its field intensity along the channel centerline. (**c**) Ion concentration and pressure changes along the centerline of the U-channel. The current numerical scenario shows a strong performance in predicting alterations in every solution variable.

2. Regression Reports

The relationship between U^*_{max} and V^* in Figure 4a has been estimated by $U^*_{max} = aV^{*n}$ at the different lateral velocities. The results are provided in Table S1.

Table S1. The regression results for $U^*_{max} = aV^{*n}$.

U_L [μm/s]	95% Confidence Interval		Predicted Value		R²	SSE
	a	n	a	n		
200	5.746-5.910	1.170-1.245	5.828	1.207	0.9999	0.00479
400	1.861-2.275	1.377-1.624	2.068	1.495	0.999	0.0379
600	0.9007-1.379	1.352-1.714	1.132	1.522	0.9978	0.08115
800	0.5702-0.9746	1.338-1.710	0.7607	1.513	0.9977	0.08727

Another regression has been used to estimate critical ζ_p in Figure 7. The corresponding results have been reported in Table S2. for three particle sizes.

Table S2. The regression results for $\zeta_p = a - (a - b)exp(-cV^*)$.

U_L [μm/s]	$d_p = 10\ [nm]$				$d_p = 100\ [nm]$				$d_p = 1000\ [nm]$				V^*
	a	b	c	ζ_{cr}	a	b	c	ζ_{cr}	a	b	c	ζ_{cr}	
200	-0.264	-39.71	3.21	-17.95	9.181	-47.05	3.22	-15.98	46.11	-46.58	1.87	-11.95	0.23
400	2.413	-134.9	1.92	-37.03	19.5	-127.9	1.48	-36.82	113.8	-123	0.75	-31.72	0.65
600	21.79	-198.9	1.04	-56.29	27.52	-198.1	0.99	-55.87	152.6	-189.1	0.52	-51.02	1.00
800	50.73	-268.7	0.69	-79.90	50.73	-268.7	0.69	-79.91	158.2	-264.6	0.47	-72.14	1.29

3. Video

Video S1. Time-dependent flow vortex behavior validation as a proof-of-concept. An experimental system in the top video is taken from [2] and the current simulation is shown in the bottom of video. The simulation and the observation are in good agreement.

References

1. Qiu, B.; Gong, L.; Li, Z.; Han, J. Electrokinetic flow in the U-shaped micro-nanochannels. *Theor. Appl. Mech. Lett.* **2019**, *9*, 36–42, doi:10.1016/j.taml.2019.01.006.
2. Sung, J.K.; Han, J. Self-sealed vertical polymeric nanoporous-junctions for high-throughput nanofluidic applications. *Anal. Chem.* **2008**, *80*, 3507–3511, doi:10.1021/ac800157q.
